# The Hospital Saturday Fund

**Published:** 1920-06-05

**Authors:** 


### THE EDITOR'S LETTER-BOX.

**Correspondence on all subjects is invited, but we cannot in any way be responsible for the opinions expressed by our correspondents, who must give their name and address as a guarantee of good faith, but not necessarily for publication. Correspondents are reminded that brevity of style and conciseness of statement greatly facilitate early insertion.**

### The Hospital Saturday Fund.

*To the Editor of* The Hospital.

Sir,—Your readers, I think, will have appreciated the courteous reply from Mr. P. A. Inman to the questions which I inflicted on the Hospital Saturday Fund on May 22. If I made no comment on its recent progress, it was only because I was considering what yet remained to be done. But it is excellent news that 60,000 subscribers have been added to the Fund since January. No less impressive are Mr. Inman's tentative figures in regard to the number of firms subscribing direct to their local hospital. The result of his complete analysis will be read with interest. From his preliminary statement in regard to thirty-one workshops, etc., it appears that nine subscribe direct in this way, three have no hospital collections, and that five subscribe alternately to the fund and to the local hospitals. Perhaps, therefore, we may infer from this preliminary statement that approximately *one-third*, mostly for an understandable reason, do *not* subscribe to the Fund.

The best news of all appears to be that a collection is made in one workshop where there are only three employees. If this number can be accepted as the smallest *qualification* for a collection, my estimate of an income of £187,500 a year for the Saturday Fund will seem to be modest enough. The map which Mr. Inman kindly enclosed can therefore be studded with further dots, so that no place with fewer than three employees is without the privilege of affiliation to a collecting committee. Would it be too much to ask for a quarterly report of the progress made toward the end which we both have in view?—Your,

Special Correspondent.

